# Supplementary material for: Elucidating ATP’s role as solubilizer of biomolecular aggregate
Source: eLife. 2024 Oct 30;13:RP99150. doi: 10.7554/eLife.99150 (PMC11524580; doi:10.7554/eLife.99150)
Supplement: Figure 1—source data 1. [file elife-99150-fig1-data1.docx]

*Ubiquitin (pdb id: 1ubq):*

MQIFVKTLT**GKT**ITLEVEPSDTIENVKAKIQDKEGIPPDQQRLIFA**GKQLEDGRT**LSDYNIQKESTLHLVLRLRGG

*Malate dehydrogenase (pdb id: 4mdh):*

SEPIRVLVTGAAGQIAYSLLYSIGNGSVF**GKDQPIILVLLDIT**PMMGVLDGVLMELQDCALPLLKDVIATDKEEIAFKDLDVAILVGSMPRRDGMERKDLLKANVKIFKCQGAALDKYAKKSVKVIVVGNPANTNCLTASKSAPSIPKENFSCLTRLDHNRAKAQIALKLGVTSDDVKNVIIWGNHSSTQYPDVNHAKVKLQAKEVGVYEAVKDDSWLKGEFITTVQQRGAAVIKARKLSSAMSAAKAICDHVRDIWFGTPEGEFVSMGIISDGNSYGVPDDLLYSFPVTIKDKTWKIVEGLPINDFSREKMDLTAKELAEEKETAFEFLSSA

*TDP-43 RRM (pdb id: 4bs2):*

**GSHMASKT**SDLIVLGLPWKTTEQDLKEYFSTFGEVLMVQVKKDLKTGHSKGFGFVRFTEYETQVKVMSQRHMIDGRWCDCKLPNSKQSQDEPLRSRKVFVGRCTEDMTEDELREFFSQYGDVMDVFIPKPFRAFAFVTFADDQIAQSLCGEDLIIKGISVHISNAEPKHNSNRQ

*Trp-cage (pdb id: 1l2y):*

NLYIQWLKDGGPSSGRPPPS

*Aβ40:*

DAEFRHDSGYEVHHQKLVFFAEDVGSNKGAIIGLMVGGVV
